# Evidence-based care of older people with suspected cognitive impairment in general practice: protocol for the IRIS cluster randomised trial

**DOI:** 10.1186/1748-5908-8-91

**Published:** 2013-08-19

**Authors:** Joanne E McKenzie, Simon D French, Denise A O’Connor, Duncan S Mortimer, Colette J Browning, Grant M Russell, Jeremy M Grimshaw, Martin P Eccles, Jill J Francis, Susan Michie, Kerry Murphy, Fiona Kossenas, Sally E Green

**Affiliations:** 1 School of Public Health and Preventive Medicine, Monash University, The Alfred Centre, 99 Commercial Road, Melbourne, Australia; 2 Centre for Health, Exercise and Sports Medicine, The University of Melbourne, Alan Gilbert Building, Level 7, 161 Barry St, Carlton, Melbourne, Australia; 3 Centre for Health Economics, Faculty of Business and Economics, Monash University, Level 2, Building 75, Monash University Clayton, Melbourne, Australia; 4 School of Primary Health Care, Monash University, Building 1, 270 Ferntree Gully Road, Notting Hill, Melbourne, Australia; 5 Clinical Epidemiology Program, Ottawa Health Research Institute, Ottawa, Canada; Department of Medicine, University of Ottawa, The Ottawa Hospital (General Campus), 501 Smyth Road, Ottawa, Canada; 6 Institute of Health and Society, Newcastle University, Baddiley Clark Building, Richardson Road, Newcastle upon Tyne, UK; 7 School of Health Sciences, City University London, Tait Building, Northampton Square, London, UK; 8 Department of Psychology, University College London, 1-19 Torrington Place, London, UK

## Abstract

**Background:**

Dementia is a common and complex condition. Evidence-based guidelines for the management of people with dementia in general practice exist; however, detection, diagnosis and disclosure of dementia have been identified as potential evidence-practice gaps. Interventions to implement guidelines into practice have had varying success. The use of theory in designing implementation interventions has been limited, but is advocated because of its potential to yield more effective interventions and aid understanding of factors modifying the magnitude of intervention effects across trials. This protocol describes methods of a randomised trial that tests a theory-informed implementation intervention that, if effective, may provide benefits for patients with dementia and their carers.

**Aims:**

This trial aims to estimate the effectiveness of a theory-informed intervention to increase GPs’ (in Victoria, Australia) adherence to a clinical guideline for the detection, diagnosis, and management of dementia in general practice, compared with providing GPs with a printed copy of the guideline. Primary objectives include testing if the intervention is effective in increasing the percentage of patients with suspected cognitive impairment who receive care consistent with two key guideline recommendations: receipt of a i) formal cognitive assessment, and ii) depression assessment using a validated scale (primary outcomes for the trial).

**Methods:**

The design is a parallel cluster randomised trial, with clusters being general practices. We aim to recruit 60 practices per group. Practices will be randomised to the intervention and control groups using restricted randomisation. Patients meeting the inclusion criteria, and GPs’ detection and diagnosis behaviours directed toward these patients, will be identified and measured via an electronic search of the medical records nine months after the start of the intervention. Practitioners in the control group will receive a printed copy of the guideline. In addition to receipt of the printed guideline, practitioners in the intervention group will be invited to participate in an interactive, opinion leader-led, educational face-to-face workshop. The theory-informed intervention aims to address identified barriers to and enablers of implementation of recommendations. Researchers responsible for identifying the cohort of patients with suspected cognitive impairment, and their detection and diagnosis outcomes, will be blind to group allocation.

**Trial registration:**

Australian New Zealand Clinical Trials Registry: ACTRN12611001032943 (date registered 28 September, 2011).

## Background

Dementia is a global problem largely driven by population ageing. A recent review of the worldwide prevalence of dementia in those aged 60 years and over found that the age standardised rates varied from 4.19% to 8.5%. In 2010, the number of people with dementia was 35.56 million, and this number is expected to increase to 115.38 million by 2050 [1]. In 2011, there were an estimated 298,000 Australians with dementia, 74% of whom were aged 75 years and older. The number of Australians with dementia is projected to reach 900,000 by 2050 [2].

Evidence-based clinical practice guidelines (CPGs) for the management of people with dementia have been published by a number of agencies, including the Scottish Intercollegiate Guidelines Network (SIGN) [3]. These guidelines include a series of evidence-based recommendations for the detection, diagnosis and management of people with dementia, both in the community and in residential care, and focus on investigations and interventions which have been shown from research to directly benefit people with dementia. We undertook a systematic search (January 2012) for clinical practice guidelines published subsequent to the SIGN guideline and identified 14, the majority of which share the same recommendations. The IRIS (Implementing Research Implementation Strategies) trial focuses primarily on detection and diagnosis recommendations from the SIGN guideline, with some adaptation for the Australian context, and two recommendations considered best practice by the IRIS clinical investigators (Table 1).

**Table 1:** Recommendations of the IRIS trial

**Recommendation**	**Details and source**
*Detection and diagnosis*	
Conduct a cognitive assessment using the Mini Mental State Examination (MMSE) in individuals with suspected cognitive impairment.	SIGN guideline (grade B recommendation*) [3].
Assess for co-morbid depression using a validated tool.	SIGN guideline (grade B recommendation*) [3]. We include the following as validated scales: Geriatric Depression Scale (GDS), Hamilton Rating Scale for Depression, and Even Briefer Assessment Scale for Depression.
Refer to Cognitive, Dementia and Memory Service (CDAMS) or specialist for access to dementia-modifying medications.	Local adaptation of SIGN guideline (grade B recommendation*) [3]. Guideline makes recommendations about specific pharmacological interventions (*e*.*g*., use of cholinesterase inhibitors). Access to dementia-modifying medication is via specialist referral in Australia.
Refer for head/brain computed tomography (CT) scan.	SIGN guideline (grade C recommendation*) [3]. Guideline recommends structural imaging. We focus only on referral for CT scan since GPs in Australia cannot refer for a MediCare rebatable magnetic resonance imaging.
Review current medication (prescription and over the counter) that may cause cognitive impairment.	Not a recommendation of the SIGN guideline. Considered best practice by the IRIS clinical investigators^†^.
Refer for pathology testing.	SIGN guideline (good practice point^‡^) [3]. Supported by other guidelines and considered best practice by the IRIS clinical investigators^†^.
*Management*	
Disclose or reinforce a diagnosis of dementia.	Not a recommendation of the SIGN guideline [3]. The SIGN guideline recommends that healthcare professionals should be aware that many people with dementia can understand their diagnosis, receive information and be involved in decision-making (grade C recommendation); that some people with dementia may not wish to know their diagnosis (grade C recommendation); and that in some situations, disclosure of a diagnosis of dementia may be inappropriate (grade D recommendation). Supported by other guidelines and considered best practice by the IRIS clinical investigators^†^.

*Grade of recommendation relates to the strength of evidence underlying the recommendation. Recommendations range from A to D, with A providing the highest level of evidence. Details of the types of evidence underlying each grade are available in the SIGN guideline [3] (pg. 2).

†Recommendation arrived at through discussion and consensus among the IRIS clinical investigators.

‡Recommended best practice based on the clinical experience of the SIGN guideline development group [3].

Detection, diagnosis and disclosure of dementia have been identified as potential evidence-practice gaps in Australian general medical practice [4,5]. Delayed diagnosis of dementia and delay in the recognition of dementia by GPs can impact outcome and restrict access to support for people with dementia and their carers. Early diagnosis can facilitate timely referral to education, counselling and support services for people with dementia and their carers, and early diagnosis is more likely to allow input from the patients about their care plans [6]. For example, there is evidence that caregiver interventions to improve well-being can delay entry to residential aged care in people with dementia [7]. Early differential diagnosis is also important in maximising the benefits of treatments and assists the patient and carer in understanding the prognosis of the disease [8]. Time from first suspicion of cognitive impairment by a GP to confirmed diagnosis or exclusion of dementia is considered too long and may take years [9]. International studies have estimated the average time from first symptoms to diagnosis, as reported by informants, to be between one and three years [10,11], with symptoms recorded in GPs’ medical records as early as five years before diagnosis [12]. A systematic review of qualitative studies suggests that diagnostic uncertainty or insufficient knowledge or experience, difficulties in disclosing the diagnosis, and the stigma attached to dementia, are barriers to diagnosis of dementia reported by primary care practitioners [13].

A limited number of randomised trials have tested the effectiveness of interventions to increase GPs’ awareness and diagnosis of people with suspected cognitive impairment and management of dementia [14-19]. These trials have evaluated a range of interventions (*e*.*g*., educational interventions, decision support software, practice-based workshops, blended learning), across different settings (United Kingdom, United States, Germany and France). The intervention effects from these trials have been mixed.

More generally, interventions designed to implement guidelines into practice have had varying success [20]. It has been suggested that this may be due, in part, to a lack of explicit rationale for the intervention choice, or the use of inappropriate methods to design the interventions [21-23]. Using theory to inform the design of interventions to implement guidelines into practice may provide a more effective approach [24]. In addition, theory provides a framework that can aid identification of factors that may modify the magnitude of intervention effects across trials [25,26]. The Theoretical Domains Framework of behaviour change provides a comprehensive framework for designing such interventions, offering broad coverage of potential change pathways [27].

A number of randomised trials are currently underway aiming to improve the management of dementia [28-31]. However, to our knowledge, no study in the Australian setting has investigated a theory-informed intervention to improve clinical practice in primary care in relation to detection and diagnosis of dementia.

### Aim and objectives

The aim of the IRIS trial is to determine if a theory-informed behaviour change intervention is effective in increasing GPs’ adherence to a clinical practice guideline for the detection, diagnosis and management of dementia in general practice (in Victoria, Australia). Our primary objectives are to establish if the intervention is effective in increasing the percentage of patients with suspected cognitive impairment who receive:

1. Cognitive assessment using the Mini Mental State Examination (MMSE); and,

2. Depression assessment using a validated scale.

These objectives reflect two key recommendations of the SIGN guideline for improving the detection and diagnosis of dementia (with level B evidence), for which there are identified evidence-practice gaps.

Secondary objectives include estimating the effects of the intervention for secondary outcomes in the categories of: GP diagnosis behaviours; proxy measures of GP diagnosis and management behaviours; and hypothesised mediators of GP behaviour (measures of motivation, capability, and opportunity to behave in a manner consistent with recommended behaviours [32]). In addition, we will conduct cost-effectiveness analyses to quantify the tradeoff between the hypothesised improvement in clinical practice and the additional costs (savings) arising from delivery of the intervention and from any subsequent changes in clinical practice and healthcare utilization within the trial period.

## Methods

The methods of the IRIS trial draw upon those of our previous implementation trials conducted in primary care settings [33,34]. At the time of submission of the trial protocol, the trial intervention has been delivered, and the baseline questionnaire measuring predictors of GPs’ diagnostic and management behaviours have been collected. Collection of patient level data has just begun (April 16).

### Trial design

The design of this trial is a parallel cluster randomised trial (C-RT) with clusters being general practices, including one or more GPs and their patients. A cluster randomised design was chosen since the intervention was targeted at GPs. Clustering at the level of the practice allows evaluation of the intervention as it would be delivered in a real world context, evaluating the direct effect of the intervention in combination with any ‘contamination’ effect arising from diffusion of the intervention amongst GPs within the same practice [35].

### Eligibility and recruitment

#### Recruitment of general practices

All GPs within the state of Victoria, Australia, listed on the Australasian Medical Publishing Company (AMPCo) database as of September 30, 2011, will be approached to participate in the trial. The AMPCo database is created from an amalgamation of sources and provides a comprehensive list of GPs in Australia. Practitioners will receive a letter of invitation, including an explanatory statement and consent form. Those who do not respond will be sent a maximum of four reminder letters, between September 2011 and February 2012. When a first GP in a practice agrees to participate, he or she will be sent invitation letters to distribute to GP colleagues who work in the same practice to facilitate more GPs to enrol per practice.

To increase awareness of the trial, notices will be placed in the newsletters of the Divisions of General Practice and Royal Australasian College of General Practitioners. Strategies to promote participation include offering continuing medical education points and an opportunity to enhance the detection and diagnosis of people with suspected cognitive impairment and their ongoing management. Practitioners will be provided with an honorarium (AUD 300) as a contribution toward practice staff time in running the electronic search of the medical records.

#### Identification of patients

Patients will be identified through an electronic search of the GPs’ medical records. We will receive de-identified data extracted from the medical records, and patients will not be contacted for any information. For these reasons, and because of the nature of the intervention (see ‘Interventions’ section), patients will not be consented to participate in the trial. This is consistent with Recommendation 3 of ‘The Ottawa Statement on the Ethical Design and Conduct of Cluster Randomized Trials’ [36], in that patients in this trial do not meet the criteria to be considered research participants.

A search module has been specifically developed as part of Pen Computer Systems (Pty Ltd) Clinical Audit Tool™ (CAT). The CAT was developed to analyse clinical information captured within general practice clinical desktop systems (*e*.*g*., Medical Director). Many Divisions of General Practice (renamed Medicare Locals in June 2012) within Victoria subscribe to the CAT, providing free access for practices within their jurisdiction. The developed module will only be activated in participating general practices, and when run, will yield a data file including all patients aged 70 years and older. The file will include demographic data (age and sex), coded diagnoses of cognitive impairment and dementia, extracted free text and dates related to cognitive and depression assessment and referral for CT scan and specialist services. To maintain anonymity of the patients, the extracted free text will be a maximum of 40 characters in length surrounding identified search terms. Search terms were compiled with input from the IRIS clinical investigators.

The search module will be run nine months after the start of the intervention (delivery of a workshop), and will search the medical records over the previous three years. From the extracted data, two cohorts of patients will be identified. The first cohort (cohort 1) will include all patients aged 70 years and older at baseline (June 22, 2012), but without a diagnosis of dementia. The second, and primary cohort of interest (cohort 2), will include the subset of cohort 1 patients for whom the GP has noted a suspicion of cognitive impairment in the medical records in the period prior to intervention delivery (prior to June 22, 2012). These patients will be identified through coded fields (*e*.*g*., coded diagnosis of cognitive impairment) and review of free text entries. Two researchers (with healthcare qualifications), who are blind to the intervention group, will independently review the free text entries. Disagreements will be resolved via discussion with a geriatrician who will not be informed of the group allocation of the patient.

The different cohorts will be used to examine GPs’ clinical behaviour with all older people (cohort 1) and with those patients whom the GP previously suspected of having cognitive impairment (cohort 2). Inclusion of the former cohort allows examination of whether the intervention is effective in raising GPs’ awareness and diagnosis of cognitive impairment and dementia in all older patients (including those with and without previously noted cognitive impairment).

#### Inclusion criteria

General practices will be included if the following criteria are met:

1. The practice is located in the state of Victoria, Australia.

2. At least one GP within the practice provides written informed consent.

3. The practice utilises a CAT-compatible general practice clinical desktop system (either Medical Director or Best Practice).

General practitioners will be included if the following criteria are met:

1. The GP works in a participating practice.

2. The GP provides written informed consent.

Patients will be included in cohort 1 if the following criteria are met:

1. The patient is ‘active’ (where ‘active’ is defined as a minimum of three visits recorded in the general practice clinical desktop system in the two-year period preceding follow-up [nine months after the start of the intervention]).

2. The patient is aged 70 years or older at baseline.

3. The patient visits the GP in the follow-up period (nine months after the start of the intervention).

Patients will be included in cohort 2 (those with suspected cognitive impairment) if any of the following additional criteria are met in the two-year period prior to the start of the intervention:

1. The patient has a coded diagnosis of cognitive impairment or free text indicating a suspicion of cognitive impairment (*e*.*g*., ‘confusion,’ ‘muddled’).

2. The patient has had an MMSE in isolation of a routine health assessment for people aged 75 years and older (75+ Health Check [37]) (an indication of the GP’s suspicion of cognitive impairment).

3. The patient has had an MMSE as part of a routine health assessment for people aged 75 years and older (75+ Health Check [37]) with a score that indicates cognitive impairment (*i*.*e*., a score between 10 and 24).

#### Exclusion criteria

General practices will be excluded if the practice principal or practice manager refuses participation.

General practitioners will be excluded if they work at more than one of the general practices included in the trial.

Patients will be excluded from both cohorts 1 and 2 if, within the two-year period prior to the start of the intervention, the patient record has a coded diagnosis of dementia or contains free text indicating dementia (*e*.*g*., Alzheimer’s, senility, dementia). Patients with dementia will be excluded since only GPs’ detection and diagnosis (not management) behaviours of dementia can be measured through patient medical records.

### Randomisation and allocation concealment

General practices meeting the inclusion criteria will be randomly allocated to the intervention or control groups. Restricted randomisation will be used to reduce the probability of baseline imbalance in factors thought to be predictive of the outcomes, and for potential gain in statistical power [38]. Four strata will be defined by geographical location of the practice (rural or metropolitan area) and the number of GPs per practice (<6 GPs and ≥6 GPs per practice). Geographical location may explain variation in some of the health service utilisation outcomes (*e*.*g*., imaging and specialist services), because of geographic proximity to services and geographic variation in socioeconomic status [39]. Cluster size (in our trial measured by the number of GPs per practice) is commonly employed as a stratification variable in cluster trials since it is often considered a proxy for characteristics of the cluster that may be predictive of the outcomes (*e*.*g*., educational environment within the practice) [35,38].

Within stratum, practices will be randomised with equal probability (1:1 ratio) to the intervention and control groups using computer-generated random numbers. Practices will be randomised at the same time by a statistician independent of the trial team. The statistician will be provided with a file containing only practice identification codes and stratification variables. Thus, the statistician will be provided with no identifying information.

### Blinding

The investigators will not be blind to group allocation since they will be involved in the delivery of the intervention. An exception to this is the trial statistician (JEM), who will be blinded to group allocation. Due to the nature of the intervention, GPs will not be blind to group allocation. General practitioners will be informed through recruitment information that they will be randomly allocated to receive access to materials about the detection, diagnosis and management of dementia, or a face-to-face workshop and access to materials. Self-report questionnaires completed by GPs will be entered by trial personnel who are blind to group allocation. Detection and diagnosis outcomes will be collected via the execution of a computer script (by general practice personnel) that extracts de-identified data from the practices’ electronic medical records.

### Interventions

#### Control group

The control group will receive a printed copy of the SIGN guideline for the management of patients with dementia [3].

#### Intervention group

In addition to receipt of the printed guideline, the GPs randomised to the intervention arm will receive an intervention designed to address the barriers to and enablers of implementation of the evidence-based recommendations. In phase one of this project, interviews were conducted with GPs in Victoria, Australia, underpinned by the Theoretical Domains Framework [40], a framework grounded in behavioural theory. The interviews were analysed using content and thematic analysis to identify the barriers and enablers relevant to each of the clinical behaviours. For example, the main factors identified as barriers to assessing cognitive function using a validated scale included negative beliefs about formal cognitive testing and the scales themselves (Beliefs about consequences); discomfort in administering the tests (Emotion); (possibly due to) limited training and confidence in using them (Skills, Beliefs about capabilities); limited access to tests or time and resources to undertake formal cognitive testing (Environmental context and resources); and patients finding testing uncomfortable or patients/family refusing testing (Social influences). The main factors enabling formal cognitive assessment included having an awareness of the need to undertake the assessment (Knowledge); possessing the necessary skills and confidence to do so (Skills; Beliefs about capabilities); and adequate time and resources (Environmental context and resources).

The intervention is an interactive, educational face-to-face workshop, led by an Australian geriatrician with expertise in dementia. The content of the workshop was designed using an intervention mapping process where the research team chose behaviour change techniques to address barriers/enablers within theoretical domains [24,41]. The behaviour change techniques delivered during the workshop will include: Information provision; Persuasive communication; Information regarding behaviour, outcome; Feedback; Social processes of encouragement, pressure, support; Self-monitoring; Modelling/ demonstration of behaviour by others; Increasing skills; Coping skills; Rehearsal of relevant skills; and Action planning. The workshop will be a combination of didactic presentations given by opinion leaders, and small group discussions led by trained facilitators.

#### Intervention fidelity

We plan to evaluate the fidelity of delivery of the intervention to assess the extent to which the intervention is delivered as planned [42]. The intervention workshops will be audio and video recorded, and these recordings will be analysed to determine which elements of the planned intervention were actually delivered.

#### Timing of recruitment, intervention delivery, and follow-up

**Figure 1:**
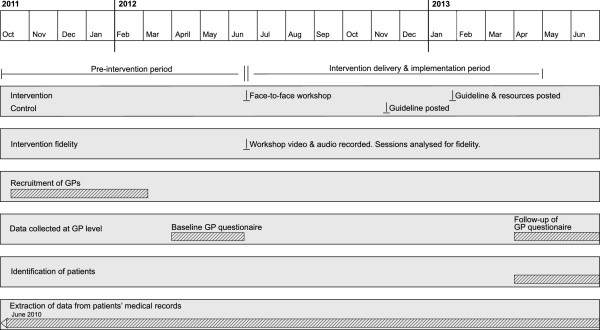
Timing of recruitment, intervention delivery, follow-up of practitioner participants and patients.

The intervention educational workshop took place on the June 23, 2012. The guidelines were sent to the control group GPs in November 2012. Questionnaires measuring predictors of GPs’ detection, diagnosis, and management behaviours were collected at baseline and will be collected nine months post the educational workshop (April 2013). The cohort of patients meeting the inclusion criteria for the trial, and GPs’ detection and diagnosis behaviours of these patients, will be identified and measured via the CAT nine months post the educational workshop (from April 2013). Figure 1 depicts the timing of recruitment, intervention delivery, and follow-up.

### Study outcomes

#### Primary outcomes

The primary outcomes (cognitive assessment using MMSE, depression assessment using validated scale) provide measures of whether the GPs undertake a formal assessment for cognitive impairment and depression (Table 2). These behaviours have been selected since they reflect the two key recommendations from the SIGN guideline [3] (with level B evidence) that have identified evidence-practice gaps and have the potential to be implemented into practice through behaviour change (*i*.*e*., there are no structural barriers to the implementation of the practice). In addition, these behaviours can be objectively measured through the CAT.

**Table 2:** Outcome measures

**Outcome**	**Data collection method**	**Outcome assessment period**	**Source**	**Level data collected**
**Primary outcomes**				
*GP behaviour*				
Cognitive assessment using MMSE^1*^	Clinical Audit Tool (CAT) electronic search	Baseline & 9 months post workshop delivery^2^	Medical record	Patient
Depression assessment using validated scale^1*^	CAT electronic search	Baseline & 9 months post workshop delivery^2^	Medical record	Patient
**Secondary outcomes**				
*GP behaviour*				
Referral to CDAMS or specialist^1†^	CAT electronic search	Baseline & 9 months post workshop delivery^2^	Medical record	Patient
Referral for CT scan^1*^	CAT electronic search	Baseline & 9 months post workshop delivery^2^	Medical record	Patient
Dementia Diagnosis^1^	CAT electronic search	Baseline & 9 months post workshop delivery^2^	Medical record	Patient
Cognitive assessment using MMSE (all patients aged 70+ years)^3*^	CAT electronic search	Baseline & 9 months post workshop delivery^2^	Medical record	Patient
Reported suspicion of cognitive impairment (all patients aged 70+ years)^3^	CAT electronic search	Baseline & 9 months post workshop delivery^2^	Medical record	Patient
Dementia diagnosis (all patients aged 70+ years)^3^	CAT electronic search	Baseline & 9 months post workshop delivery^2^	Medical record	Patient
*Proxy measures of GP behaviour*				
Self-report of adherence to recommended behaviours:	Questionnaire	Baseline & 9 months post workshop delivery	Practitioner	Practitioner
Cognitive assessment using MMSE*	(1 item each)			
Depression assessment using validated scale*				
Referral to CDAMS or specialist^†^				
Referral for CT scan*				
Review of medications^§^				
Ordering of pathology tests^‡§^				
Behavioural simulation to adhere to recommended behaviours:	Questionnaire (clinical vignettes)	9 months post workshop delivery	Practitioner	Practitioner
Cognitive assessment using MMSE*				
Depression assessment using validated scale*				
Referral to CDAMS or specialist†				
Referral for CT scan*				
Review of medications§				
Ordering of pathology tests‡§				
Disclosure of diagnosis to patient‡§				
Disclosure of diagnosis to carer‡§				
*Hypothesised mediators of GP behaviour*				
Intention to adhere to recommended behaviours:	Questionnaire	Baseline & 9 months post workshop delivery	Practitioner	Practitioner
Cognitive assessment using MMSE*	(3 items)			
Depression assessment using validated scale*	(3 items)			
Disclosure of diagnosis to patient‡§	(6 items)			
Disclosure of diagnosis to carer‡§	(2 items)			
Behavioural constructs for primary outcomes^4^	Questionnaire	Baseline & 9 months post workshop delivery	Practitioner	Practitioner
	(47 items)			

Symbols indicate source of recommended behaviour: * SIGN guideline; † Local adaptation of SIGN guideline (by IRIS clinical investigators); ‡ Other guidelines; § Considered best practice by the IRIS clinical investigators.

^1^Active patients aged 70 years and over in whom the GP suspects cognitive impairment at baseline (cohort 2). Active is defined as a minimum of three visits recorded in the general practice clinical desktop system in the two-year period preceding follow-up (nine months post workshop delivery)).

^2^For this variable, the outcome is measured over the two-year period prior to randomisation and nine months post workshop delivery.

^3^Active patients aged 70 years and older (cohort 1). See footnote 1 for the definition of active.

^4^Table 3 provides details of behavioural construct domains for the primary outcomes.

#### Secondary outcomes

##### GP behaviour

The secondary outcomes include measures of GPs’ diagnostic behaviours (Table 2). The chosen behaviours are recommendations from the SIGN guideline [3], including some adaptation for the Australian context (*e*.*g*., referral to CDAMS, details of adaptation available in Table 1). Two behaviours, ‘review of medications’ and ‘ordering of pathology tests’ are not recommendations of the SIGN guideline, but were considered best practice by the IRIS clinical investigators (Table 1). We have also included outcomes (dementia diagnosis, reported suspicion of cognitive impairment) measuring whether the intervention is effective in raising GPs’ awareness and diagnosis of cognitive impairment and dementia for all patients aged 70 years and older (cohort 1).

##### Proxy measures of GP behaviour

Proxy measures of all GP diagnostic behaviours have been included. For some diagnostic behaviours (referral to specialist, review of medications, ordering of pathology tests), it is not possible to use the CAT. Proxy measures provide an alternative method for measuring behaviour in such circumstances, and there is some evidence showing they are predictive of behaviour [43]. We have included proxy measures for the two management behaviours, disclosure of diagnosis of dementia to (i) patients and (ii) carers. While disclosure of diagnosis is not a recommendation of the SIGN guideline, there are many ethical arguments favouring disclosure [44], and the IRIS clinical investigators strongly advocated for disclosure. Furthermore, disclosure was identified as salient in the interviews with GPs in phase I of this project.

##### Mediators of GP behaviour

**Figure 2:**
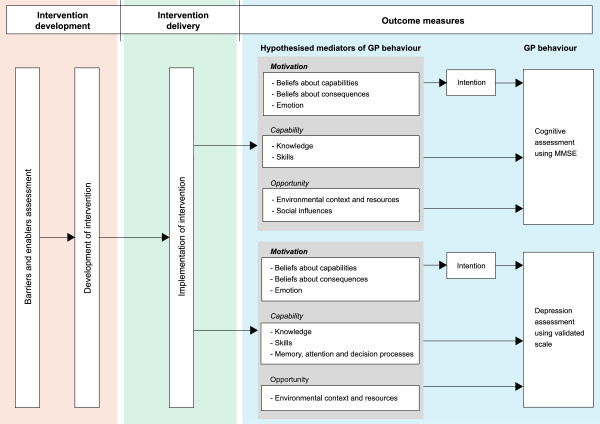
Hypothesised causal pathway model for the primary outcomes.

For the two key recommendations (undertaking a formal assessment for cognitive impairment and for depression), potential mediators of GP behaviour include measures of behavioural constructs (*e*.*g*., emotion, knowledge, skills, and social influences) (Table 3). These mediators reflect the barriers and enablers that were identified in phase I of this project (through interviews with GPs), and were targeted through the intervention components. We include measures of intention to adhere to the two key recommendations, since intention in many theories is considered the most immediate predictor of behaviour [45], and we hypothesise that intention will mediate the relationship between GPs’ motivation and behaviour. If the intervention is effective, we posit that differences in these mediators between groups will be observed. Figure 2 displays the causal pathway demonstrating the hypothesised relationship between the intervention, mediators and behaviour. Examination of the effects of the intervention along the causal pathway has been restricted to the primary outcomes to limit respondent burden.

**Table 3:** Behavioural construct domains (hypothesised mediators of GP behaviour)

**Domains**	**Domain definitions **[27]** (adapted from Michie *et al*. **[40]**)**	**Domain measured for behaviour**	
		Cognitive assessment^1^	Depression assessment^2^
*Motivation*			
Intention^3^	A conscious decision to perform a behaviour or a resolve to act in a certain way	✓	✓
Beliefs about capabilities	Acceptance of the truth, reality or validity about an ability, talent or facility that a person can put to constructive use	✓	✓
Beliefs about consequences	Acceptance of the truth, reality or validity about outcomes of a behaviour in a given situation	✓	✓
Emotion	A complex reaction pattern, involving experiential, behavioural and physiological elements, by which the individual attempts to deal with a personally significant matter or event	✓	✓
*Capability*			
Knowledge	An awareness of the existence of something	✓	✓
Skills	An ability or proficiency acquired through practice	✓	✓
Memory, attention and decision processes	The ability to retain information, focus selectively on aspects of the environment, and choose between two or more alternatives	✗	✓
*Opportunity*			
Environmental context and resources	Any circumstance of a person’s situation or environment that discourages or encourages the development of skills and abilities, independence, social competence, and adaptive behaviour	✓	✓
Social influences	Those interpersonal processes that can cause individuals to change their thoughts, feelings or behaviours	✓	✗

^1^Cognitive assessment using MMSE. Three items per behavioural construct domain, except for Beliefs about consequences, which is four items; providing a total of 25 items.

^2^Depression assessment using validated scale. Three items per behavioural construct domain, except for Beliefs about consequences, which is four items; providing a total of 25 items.

^3^Intention referred to as ‘Motivation and goals’ in the Theoretical Domains Framework [40].

We have included measures of intention for the two management behaviours, disclosure of diagnosis of dementia to (i) patients and (ii) carers.

### Outcome measurement

Table 4 provides a summary of the measurement tools. In brief, GPs’ detection and diagnostic behaviours will be measured through the CAT where possible. Proxy measures and mediators of GP behaviour will be measured through a paper-based questionnaire (available in Additional file 1 - IRIS behavioural construct questionnaire).

**Table 4:** Summary of measurement tools

**Outcome or category of outcomes**	**Measurement tool**	**Details**
*GP behaviour*		
Cognitive assessment using MMSE	CAT electronic search^†^	MMSE results recorded in the patient file or free text indicates an MMSE has been undertaken.
Depression assessment using validated scale	CAT electronic search^†^	Geriatric Depression Scale (GDS) results recorded in the patient file or free text indicates GDS, Hamilton Rating Scale for Depression, Even Briefer Assessment Scale for Depression has been undertaken.
Referral to CDAMS or specialist	CAT electronic search^†^	Free text indicates that the patient has been referred to CDAMS, ACAS (Aged Care Assessment Service), or a geriatrician.
Referral for CT scan	CAT electronic search^†^	CT scan has been requested or free text indicates that a CT (head) scan has been requested/undertaken.
Dementia diagnosis	CAT electronic search^†^	Coded diagnosis of dementia or free text indicates that the patient has dementia or Alzheimer’s disease.
Cognitive assessment using MMSE (all patients aged 70+ years)	CAT electronic search^†^	MMSE results recorded in the patient file or free text indicates an MMSE has been undertaken.
Reported suspicion of cognitive impairment (all patients aged 70+ years)	CAT electronic search^†^	Coded diagnosis of cognitive impairment or free text indicates a suspicion of cognitive impairment (*e*.*g*., confusion, muddled, cognitive), or; MMSE undertaken in isolation of the 75+ Health Check (an indication of GP’s suspicion of cognitive impairment), or; MMSE undertaken as part of 75+ Health Check with score indicating cognitive impairment (*i*.*e*., a score between 10 and 24).
Dementia diagnosis (all patients aged 70+ years)	CAT electronic search^†^	Coded diagnosis of dementia or free text indicates that the patient has dementia or Alzheimer’s disease.
*Proxy measures of GP behaviour*		
Self-report of adherence to recommended behaviours (*e*.*g*., Cognitive assessment using MMSE)	Questionnaire* (1 item per behaviour)	Adapted from Eccles *et al*. [45]. Example item: ‘Thinking about the last 10 patients you saw who you suspected had cognitive impairment, how many of them did you assess for cognitive function using the Mini Mental State Examination (MMSE)?’
Behavioural simulation to adhere to recommended behaviours (e.g., Cognitive assessment using MMSE)	Questionnaire (6 clinical vignettes)	Vignettes simulate clinical decision-making about detection, diagnosis and management of dementia. Vignettes include a range of clinical variables: sex, age (72 – 88 years), cognitive function (including changes to memory, personality, behaviour, cognition), depression, and other elements. These clinical variables were drawn from previously published vignettes [46-52] and from the experience of the clinical investigators. The vignettes, and response options, will be piloted with two to three GPs prior to being administered.
*Hypothesised mediators of GP behaviour*		
Intention to adhere to recommended behaviours (*e*.*g*., Cognitive assessment using MMSE)	Questionnaire* (3 items per behaviour)	Adapted from Eccles *et al*. [45], Francis *et al*. [53],and Foy *et al*. [54]. Items for Cognitive assessment using MMSE include ‘I would make it a high priority to use the MMSE to assess the cognitive function of these patients,’ ‘I plan to use the MMSE to assess the cognitive function of these patients,’ ‘I intend to use the MMSE to assess the cognitive function of these patients.’ Each item measured on a 7-point Likert scale ranging from strongly disagree to strongly agree (1 to 7). Scores are then averaged to create a behavioural intention score. Higher scores reflect greater intention to assess cognitive function using the MMSE.
Behavioural constructs for primary outcomes	Questionnaire*	Adapted from [34]. There are 49 items in total. Example of the items used to measure various domains (noted in brackets) for the behaviour depression assessment using a validated scale include: ‘How much do you know about validated scales for assessing depression in these patients?’ (knowledge), ‘Using a validated scale to assess these patients for depression is sometimes stressful’ (emotion), ‘Lack of time may prevent me from using a validated scale to assess these patients for depression’ (environmental context and resources). Each item is measured on a 7-point Likert scale. All constructs are measured using three items (which are averaged to create a final score for the construct), except beliefs about consequences, which is measured using four items.

^*^Questionnaire available in Additional file 1 – IRIS Behavioural construct questionnaire. ^†^ Two researchers (with healthcare qualifications), who are blind to intervention group, will independently review the free text entries to decide if the behaviour has occurred. Disagreements will be resolved via discussion with a geriatrician who will not be informed of the group allocation of the patient.

### Data quality assurance

GP questionnaires will be checked for errors and missing data as they are returned, and GPs will be followed up to clarify anomalies. Double data entry will be used to enter GP paper-based questionnaires. Inconsistencies will be investigated by referring back to the paper-based version. Non-responding GPs will be contacted by phone to encourage completion of the questionnaire.

Free text entries extracted from the CAT contain a maximum of 40 characters surrounding the identified search term, to maintain anonymity of the patients. The short length of these text extracts is likely to lead to difficulties in coding variables for some patients. Therefore, two researchers, who are blind to intervention group, will independently review the text extracts. To improve consistency in coding between researchers, a coding dictionary will initially be created from a sample of text extracts. Disagreements will be resolved via discussion with a geriatrician who will not be informed of the group allocation of the patient.

### Sample size

The primary outcomes of the IRIS trial include cognitive assessment using MMSE and depression assessment using a validated scale in patients with suspected cognitive impairment (cohort 2). The trial has been powered to detect a difference of 15% in rates of the behaviours between groups (assuming control group rates of 50%). Using sample size formula (2) of Eldridge *et al*. [55] and assuming, on average 20 patients per practice are identified, with a coefficient of variation in practice size of 0.7, and an intra-cluster correlation of 0.10, 45 practices per group will be sufficient to detect the 15% increase in recommended behaviours with 90% power (two-sided significance level of 5%). Allowing for 25% attrition in practices, we aim to recruit 60 practices per group. Justifications of the parameters used in the sample size calculation are available in Additional file 2 – IRIS sample size calculations.

### Effectiveness analyses

#### Analysis subsets

Intention-to-treat (ITT) analyses are generally recommended in randomised trials for the primary reason of preserving the benefits of randomisation; namely, maintaining the comparability of the intervention groups in known and unknown prognostic factors [56]. In addition, it has been argued that ITT analyses compared with other analysis strategies (*e*.*g*., per-protocol) are more appropriate for pragmatic trials since they provide estimates of intervention effect that are more reflective of what would be observed if the intervention was implemented in routine clinical practice [57-59].

While requirements of an ideal ITT analysis (including compliance with the randomised intervention, no missing responses, and follow-up on all participants [57]) have been established for patient randomised trials, only recently has there been more detailed discussion of the definition and application of ITT analyses in C-RTs [60]. In C-RTs with adequate allocation concealment, comparability of intervention groups can be compromised not only through missing responses and loss to follow-up (as occurs in patient randomised trials), but also through recruitment of participants occurring post randomisation. Furthermore, loss to follow-up in cluster trials can occur at different levels (clusters and patients) because of the hierarchical structure of the design.

In the IRIS trial, retrospective identification of eligible participants will be undertaken (post randomisation) by researchers blind to group allocation, so the potential for selection bias will be minimised. In addition, bias arising from missing responses and loss to follow-up at the patient level will be minimal, since data will be extracted through the CAT on all eligible patients. However, practices and GPs may withdraw prior to data being extracted on their patients, resulting in empty clusters. A full application of the ITT principle in this circumstance would require the empty clusters to be accounted for in the analysis. Accounting for empty clusters would require strong assumptions to be made about patient characteristics and outcomes based on GP or cluster characteristics.

We therefore plan to present a modified ITT analysis as our primary analysis, where we will analyse clusters, GPs and patients, as they have been randomised, regardless of the intervention they have received, but will not impute missing data. As part of the secondary analyses, we will attempt to examine the potential impact of empty clusters on the intervention effects for the primary outcomes. Reasons for practice and GP withdrawal will be collected, and even in the circumstance of withdrawal, we will seek permission to run the CAT to extract data on patients.

#### Descriptive analyses at baseline

Descriptive statistics of baseline demographic and potential confounding variables at the patient, GP, and practice level will be presented (Table 5). These statistics will allow assessment of the comparability of intervention groups at baseline, and provide descriptive information about the study sample.

**Table 5:** Baseline characteristics at patient, GP, and practice level (presented by intervention group)

**Patient level**	**GP level**	**Practice level**
Cohort 1*	Age (years) (mean, SD)	No. GPs per practice (mean, SD)
Age (years) (mean, SD)	Sex (no., % female)	Rural practices (no., %)
Sex (no., % female)	No. of years since graduated from medical school (mean, SD)	Estimated total number of patients on the practice’s books (mean, SD)
Suspected cognitive impairment (no., %)	Country of medical training (Australia or overseas) (no., % Australia)	Practice nurse available (no., %)
		Involved in undertaking health assessments for people aged ≥75 years (no., %)
Cognitive assessment using MMSE (no., %)	Yrs. practised in Aust. if overseas medical training (mean, SD)	
		Undertakes full assessment or part (in combination with GP) (no., %full)
Cohort 2*	GP registrar (no., %)	Involved in other aged care activities (no., %)
Age (years) (mean, SD)	Fellow of RACGP (no., %)	Other health practitioners work in the practice (specialist, allied health) (no., %)
Sex (no., % female)	Member of GP Division in their region (no., %)	Practice formally involved in training GP registrars (no., %)
Cognitive assessment using MMSE (no., %)	Hours spent per week in clinical practice (mean, SD)	Practice services residential care facilities (no., %)
Depression assessment using validated scale (no., %)	No. patients seen per week (mean, SD)	Method of billing (bulk bill or co-payment) (no., %bulk bill)
	Percentage of patients over 70 (mean, SD)	
		Age of practice (years) (mean, SD)
Referral to CDAMS or specialist (no., %)	Special interest in dementia (no., %)	Ownership (corporate or privately owned) (no., %corporate)
Referral for CT scan (no., %)	Special interest in aged care (no., %)	
	Self-report of adherence to recommended behaviours (mean, SD)	
	Intention to adhere to recommended behaviours (mean, SD)	
	Behavioural constructs for primary outcomes (mean, SD)	

^*^Cohort 1: Active patients aged 70 years and older at baseline. Cohort 2: Active patients aged 70 years and older in whom the GP suspects cognitive impairment at baseline.

#### Primary analyses

Marginal models using generalised estimating equations (GEEs) will be fitted for binary outcomes (using a logit link function) to estimate the effectiveness of the intervention. These models appropriately account for the correlation of responses within practice. We will assume an exchangeable correlation structure (where responses from the same practice are assumed to be equally correlated [35,61]) and use robust variance estimation (which yield valid standard errors of the intervention effect even if the within-cluster correlation structure has been misspecified [62,63]). Generalised estimating equations do not constrain ICCs to be positive; however, in the context of this trial, the likely explanation for a negative ICC is sampling variability, and not a true underlying negative ICC [35,64]. Therefore, in the event that the ICC from a particular analysis is negative, we will estimate the intervention effect using ordinary logistic regression, which will yield conservative estimates of standard errors.

The measure of intervention effect arising from the above models is an odds ratio. To aid interpretation, we plan to also present risk differences [65]. Risk differences will be calculated from marginal probabilities estimated from the fitted models [66]. Confidence intervals for the risk differences will be calculated using bootstrap methods, appropriately allowing for the clustered structure of the data.

Linear mixed models (LMM) will be fitted for continuous outcomes to estimate the effectiveness of the intervention, allowing for clustering with a random practice effect [67]. Continuous outcomes will only be measured at the level of the GP (self-report measures, intention, and behavioural constructs) and there are likely to be few practices with multiple GPs. In circumstances where there are a small proportion of clusters with multiple observations, LMM have been shown to perform slightly better than GEEs [68]. For skewed continuous outcomes, model-based standard errors will be compared with those obtained from bootstrapping.

**Figure 3:**
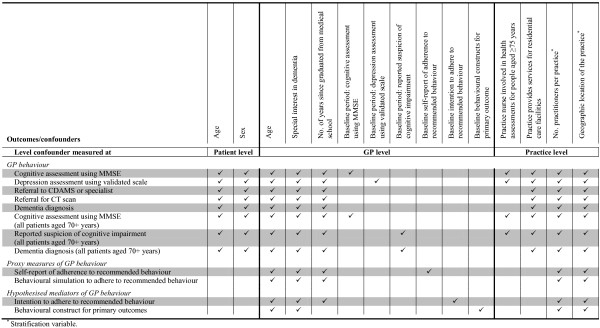
Potential confounding variables adjusted for in the primary analyses.

Our primary analyses of outcomes will include adjustment for the stratification variables (*e*.*g*., geographical location of the practice, number of GPs per practice) and pre-specified potential confounding variables (Figure 3). The potential confounders have been selected through discussion with the investigators and examination of the confounders adjusted for in other similar implementation trials (*e*.*g*., [16,30]). All pre-specified confounders will be included in the models even when no baseline imbalance exists, since confounder selection strategies based on observed data (*e*.*g*., selecting confounders using preliminary statistical tests) result in models with poor statistical properties (*e*.*g*., incorrect type I error rates) [69-72]. If there are outcomes with limited data or events, we will only adjust for the stratification variables and, where appropriate, the baseline of the outcome variable (*e*.*g*., self-report measures, intention, and behavioural constructs). For each outcome, the estimate of intervention effect and its 95% CI will be provided. For primary outcomes, we plan to provide estimates of ICCs and their 95% CI.

Regression diagnostics will be used to assess the influence of outliers on estimates of intervention effect and for analysing residuals. No adjustment will be made for multiple testing. All tests will be two-sided and carried out at the 5% level of significance.

#### Secondary analyses

GEEs fitted to binary outcomes yield unbiased estimates of intervention effect only when data are missing completely at random [73]. As noted previously (in the ‘Analysis subsets’ section), empty clusters arising from practices or GPs withdrawing post randomisation but prior to extraction of patient data may occur, and this may introduce bias. We will attempt to examine the potential impact of empty clusters on the intervention effects for the primary outcomes using weights to allow for patterns of ‘missingness’ [74]. Weights will be created based on proxy measures of clinical behaviour for the key recommendations (*e*.*g*., self-report adherence to cognitive assessment using MMSE, intention to adhere to cognitive assessment using MMSE).

Two inclusion criteria used to define cohort 2 (patients with suspected cognitive impairment) are based on use of the MMSE in the baseline period. If GPs suspect cognitive impairment, patients should receive further assessments using the MMSE, regardless of whether they have been previously assessed. The rate of MMSE assessment is likely to be higher in patients who have received a previous MMSE, since their GP is already more likely to adhere to this recommendation. Consequently, an MMSE assessment in the baseline period may modify the intervention effect for the primary outcome of cognitive assessment using MMSE. We will examine this by fitting a model that includes an interaction term between the intervention group and an administration of the MMSE in the baseline period.

For the primary outcomes, we plan to undertake a per-protocol analysis to estimate the effect of the intervention for the subgroup of GPs who comply with the intervention. Compliance to the intervention is defined as attendance at the workshop.

We plan to undertake explanatory analyses for the primary outcomes to examine if intervention effects are explained by our hypothesized mediators of practitioner behaviour (Figure 2) [75-78]. These explanatory analyses will form a separate publication.

In the IRIS trial, some behaviours will be measured both objectively (through medical records) and subjectively (self-report and behavioural simulation). When objective measures are available, we will examine the predictive validity of the subjective measures; this will aid in interpretation of the subjective measures and contribute evidence of their measurement properties.

### Economic evaluation

Several recent studies have estimated the costs and benefits of early diagnosis and timely intervention for dementia. Banerjee and Wittenberg (2009) conducted a modelled cost-utility analysis comparing the use of multidisciplinary, interagency teams for the early diagnosis and treatment of dementia (Croydon Memory Service Model) against usual care in England. Results from this analysis suggested that ‘…a gain of between 0.01 and 0.02 QALYs per person year …plus a 10% diversion of people with dementia from residential care …would be sufficient to render the service cost-effective (in terms of positive net present value)’ [79]. While Banerjee and Wittenberg suggested that such improvements ‘seem very likely to be achievable,’ the available evidence for the effectiveness of the Croydon Memory Service Model is limited and subject to a high risk of bias [80]. Wolfs *et al*. conducted cost utility and cost effectiveness analyses alongside the Maastricht Evaluation of a Diagnostic Intervention for Cognitively Impaired Elderly (MEDICIE) C-RT to compare diagnosis and intervention through the Diagnostic Observation Centre for Psycho-Geriatric Patients (DOC-PG) against usual care in the Netherlands. Results from the cost-utility analysis suggested that the DOC-PG yields an average gain of 0.05 of a QALY over usual care at an average incremental cost of just €65 (€1267 per QALY gained). While the probability that the DOC-PG was cost-effective exceeded 50% at a funding threshold of €20,000 per QALY, there remained a 20% probability that usual care is more cost-effective than the DOC-PG even at a threshold of €80,000 [81].

The interventions evaluated in each of these previous studies entail care outside of general practice by, for example, commissioning ‘a new service to work in a complementary way with existing primary and secondary care services’ [81]. Treatment effects are achieved via a change in practitioner rather than a change in clinical practice (behaviour change). Such an approach may not be suited to all settings. The economic evaluation to be conducted alongside the IRIS trial will be the first to estimate the costs and benefits associated with changing clinical practice within the existing and dominant model of primary care in Australia to improve the adherence of general practitioners to recommended behaviours for the detection and diagnosis of dementia.

Specifically, cost effectiveness analyses will be conducted alongside the IRIS C-RT to quantify the additional costs (savings) and improvements in adherence to the CPG arising from delivery of the IRIS implementation intervention, compared with passive dissemination of the CPG. Evaluation of costs and health gains arising from delivery of the intervention (*ex post* of development of the implementation intervention) will be informative to policy-makers and hospital administrators considering a wider roll-out of the IRIS implementation intervention [82]. Secondary aims will be to determine whether the incremental treatment costs of the IRIS intervention are offset by reductions in health service expenditure within the trial period (*i*.*e*., whether implementation is cost-saving as compared with existing practice), and to determine whether the IRIS intervention dominates existing practice (*i*.*e*., less costly but no less effective). The time horizons for inclusion of relevant costs and consequences for the trial-based evaluation described here will be limited to the period of follow-up of participants in cohorts 1 and 2 (nine months post-delivery of the intervention).

The economic evaluation alongside the IRIS C-RT will take a health sector perspective in identifying, measuring and valuing costs and consequences within the time horizon. The time horizon for the IRIS trial necessarily excludes costs and consequences beyond the short-run effects observable in the trial excepting insofar as they are reflected in adherence to the key-recommendations of the CPG. In addition, we exclude some dimensions of adherence not captured by the primary effectiveness outcomes. Research and evaluation costs will be excluded except where they might plausibly contribute to a clinically significant treatment effect. Costs common and invariant to both intervention and control groups (*e*.*g*., costs associated with development and standard dissemination of the guideline) will not be explicitly calculated for the incremental analysis described here. Finally, some cost categories unlikely to produce clinically and economically significant variation in incremental cost will be excluded (*e*.*g*., opportunity cost of patient time while attending treatment) to simplify our analysis [83].

Additional methods for the economic evaluation alongside the IRIS trial including methods for the identification, measurement and valuation of outcomes and resource use are described in Additional file 3 – IRIS additional methods for the economic evaluation. Results from the economic evaluation alongside the IRIS trial will be expressed as: additional costs (savings) per additional patient assessed using MMSE and additional costs (savings) per additional patient receiving depression assessment using validated scale.

### Publication policy

The results from the trial will be published regardless of the outcome. Reporting of this trial will adhere to the relevant, and most up-to-date, CONSORT (Consolidated Standards of Reporting Trials) statement [84] and its relevant extensions [65,85,86].

### Ethical review

Ethical approval for this trial was obtained from the Monash University Human Research Ethics Committee (CF11/0727 – 2011000192, CF09/3631 - 2009001968). The investigators will ensure that the trial is conducted in compliance with this protocol and the Australian National Statement on Ethical Conduct in Human Research [87].

## Competing interests

MPE is editor-in-chief, SM and DOC are associate editors, and JMG is a member of the Senior Advisory Board of Implementation Science. Editorial decisions regarding publication of this manuscript were made independently by another editor. The remaining authors declare they have no competing interests.

## Authors’ contributions

SEG, JEM, SDF, DAO, DSM, and CJB conceptualised and secured funding for the trial; SEG was the lead investigator of the funding application. SEG, JEM, SDF, DAO, DSM, CJB, GMR, JMG, MPE, JJF, SM, KM, and FK designed the trial. SEG, SDF, DAO, JJF, SM, and GMR were involved in the design or delivery of the intervention, or both. JEM wrote the first draft of this publication, with contributions from SDF (the ‘Interventions’ section) and DM (the ‘Economic evaluation’ section). All authors contributed to revisions of the manuscript and read and approved the final manuscript.

## Supplementary Material

Additional file 1 **IRIS behavioural construct questionnaire.** This file includes the behavioural construct questionnaire. Click here for file

Additional file 2 **IRIS sample size calculations.** This file provides details of the sample size calculations used in IRIS. Click here for file

Additional file 3 **IRIS additional methods for the economic evaluation.** This file provides additional details of the methods used for the economic evaluation alongside the IRIS trial. Click here for file
