# Supplementary material for: Evidence-based care of older people with suspected cognitive impairment in general practice: protocol for the IRIS cluster randomised trial
Source: Implement Sci. 2013 Aug 19;8:91. doi: 10.1186/1748-5908-8-91 (PMC3765181; doi:10.1186/1748-5908-8-91)
Supplement: Additional file 1 — IRIS behavioural construct questionnaire. This file includes the behavioural construct questionnaire. [file 1748-5908-8-91-S1.docx]

|  |
| --- |

**
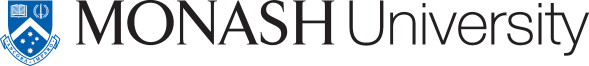
**

**IRIS Study**

**Investigating Research Implementation Strategies for care of people with suspected cognitive impairment**

**Questionnaire for General Practitioners**

Thank you for agreeing to participate in the IRIS study and for completing this baseline survey.

This survey explores factors that influence how general practitioners manage patients 70 years and over with suspected cognitive impairment and dementia. The survey focuses on procedures undertaken in the diagnostic work-up of these patients and whether general practitioners tell patients with dementia their diagnosis.

Please read each question carefully. We are interested in your opinions and there are no correct or incorrect responses. Some of the questions may seem repetitive or similar, but we do ask that you try to answer each question as best you can (as we have included several which are subtly different to survey shades of opinion).

The survey should take approximately 20 minutes to complete.

All information that you provide will be confidential**.** No information that could lead to the identification of any individuals will be disclosed in any reports or to any other party.

Please return the survey in the reply paid envelope provided.

**Thank you very much for your participation**

Professor Sally Green

on behalf of the IRIS Study Team

School of Public Health and Preventive Medicine, Monash University

If you have any questions or would like more information please contact myself or

The IRIS Study Team, School of Public Health and Preventive Medicine

T: (03) 9903 0366

E: [iris@med.monash.edu](mailto:iris@med.monash.edu)

**THIS PAGE HAS BEEN LEFT INTENTIONALLY BLANK**

**PART A – Demographic questions**

Please answer the following questions about you.

|  |  | |  |  | | | | | |  | | | | |  |  |  |  |  |  |  |  |  |  |  |  |  |  |  |  |  |  |  |
| --- | --- | --- | --- | --- | --- | --- | --- | --- | --- | --- | --- | --- | --- | --- | --- | --- | --- | --- | --- | --- | --- | --- | --- | --- | --- | --- | --- | --- | --- | --- | --- | --- | --- |
| **1** | First name: | | | | Surname: | | |  | | | | |  |  |  |  |  |  |  |  |  |  |  |  |  |  |  |  |  |  |  |  |  |
| **2** | What year were you born? [*please specify*] | | | | \| 1 \| 9 \|  \|  \| \| --- \| --- \| --- \| --- \| | | |  |  |  |  |  |  |  |  |  |  |  |  |  |  |  |  |  |  |  |  |  |  |  |  |  |  |
| **3** | Are you male or female? [*please tick one box*] | | | | ❑ Male ❑ Female | | |  | | |  |  |  |  |  |  |  |  |  |  |  |  |  |  |  |  |  |  |  |  |  |  |  |
| **4** | What year did you graduate from medical school? [*please specify*] | | | | \|  \|  \|  \|  \| \| --- \| --- \| --- \| --- \| | | |  |  |  |  |  |  |  |  |  |  |  |  |  |  |  |  |  |  |  |  |  |  |  |  |  |  |
| **5** | Where was your medical training conducted? *[please tick one box]* | | | | ❑ Australia ❑ Overseas | | |  | | | | | | | |  |  |  |  |  |  |  |  |  |  |  |  |  |  |  |  |  |  |
| **5a** | | If overseas, how many years have you practised in Australia? [*please specify*] | | | \|  \|  \| \| --- \| --- \|   year(s) | | |  |  |  |  |  |  |  |  |  |  |  |  |  |  |  |  |  |  |  |  |  |  |  |  |  |  |
| **6** | Are you currently a GP registrar?  [*please tick one box*] | | | | ❑ Yes ❑ No | | |  | |  | | | | |  | | | | |  | | |  | | |  | | |  |  |  |  |  |
| **7** | Are you a Fellow of the RACGP?  [*please tick one box*] | | | | ❑ Yes ❑ No | | |  | |  | | | | |  | | | | |  | | |  | | |  | | |  |  |  |  |  |
| **8** | RACGP No. | | | | ACRRM No. | | |  |  |  |  |  |  |  |  |  |  |  |  |  |  |  |  |  |  |  |  |  |  |  |  |  |  |
| **9** | Are you a member of the GP Division in your region? [*please tick one box*] | | | | ❑ Yes ❑ No | | |  | |  | | | | |  | | | | |  | | |  | | |  | | |  |  |  |  |  |
| **10** | In a typical week, how many **hours** do  you spend in clinical practice? [*Include hours worked in direct patient care plus other clinical activities*] | | | | hours | | |  | |  | | | | |  | | | | |  | | |  | | |  | | |  |  |  |  |  |
| **11** | In a typical week, how many patients do you see? [*please specify*] | | | |  | | |  | | | |  | | | | | |  |  | | | | | |  | |  |  |  |  |  |  |  |
| **12** | Approximately, what percentage of your patients are over 70? [*please specify*] | | | | \|  \|  \|  \| \| --- \| --- \| --- \|   % of my patients are over 70 years old | | |  |  |  |  |  |  |  |  |  |  |  |  |  |  |  |  |  |  |  |  |  |  |  |  |  |  |
| **13** | Do you have a special area of interest in your practice? [*please tick all that apply*]  ❑ None  ❑ Dementia  ❑ Aged care    ❑ Other [*please specify*]: | | | |  | | |  |  |  |  |  |  |  |  |  |  |  |  |  |  |  |  |  |  |  |  |  |  |  |  |  |  |
| **14** | Have you undertaken any formal Postgraduate training relevant to the management of dementia, for example, short courses, postgraduate diploma or Master degree? [*please tick one box*] | | | | | ❑ Yes ❑ No | | |  | | |  | | | | |  | | | |  | | |  | | | |  | | |  |  |  |
| **15** | Have you undertaken any CME or postgraduate training on dementia in the last 12 months? [*please tick one box*] | | | | | | ❑ Yes ❑ No |  | | | | | |  | | | | | | | |  | | | | | | | |  | |  |  |
| **16** | Do you work in more than one general practice? [*please tick one box*] | | | | ❑ Yes ❑ No | | | |  | | |  | | | | |  | | | |  | | |  | | | |  | | |  |  |  |

**16a** If you do work in more than one practice, please provide details of the practices you work in:

| **Other practice details** |
| --- |
| Practice name: _________________________________________________________________  Street number & name: ___________________________________________________________  Suburb:______________________________ State:_____________ Postcode:_____________  Practice phone:__________________________ Practice fax: __________________________ |
|  |
| **Other practice details** |
| Practice name: _________________________________________________________________  Street number & name: ___________________________________________________________  Suburb:______________________________ State:_____________ Postcode:_____________  Practice phone:__________________________ Practice fax: __________________________ |

If you work in more than two practices, please write details below:

**PART B – Questions about clinical management**

The questions in this section all relate to **your clinical management of patients 70 years and over who you suspect have cognitive impairment.**

Please respond to each question below by **writing a number between 0 and 10** which best reflects your current practice (or that of staff within your practice who undertake the specified procedure(s) at your instigation or on your behalf).

|  | | | |  |  |  |
| --- | --- | --- | --- | --- | --- | --- |
| **Thinking about the last 10 patients, 70 years and over, you saw who you suspected had cognitive impairment, how many of them did you...... :** *(specify the number between 0 and 10 for each item below)* | | | |  |  |  |
|  | 1. Assess for cognitive function using clinical interview? |  | **…of 10** [*please specify*] |  |  |  |
|  | 1. Assess for cognitive function using the Mini Mental State Examination (MMSE)? |  | **…of 10** [*please specify*] |  |  |  |
|  | 1. Assess for co-morbid depression using clinical interview? |  | **…of 10** [*please specify*] |  |  |  |
|  | 1. Assess for depression using a validated scale**¹** ? (*see footnote*) |  | **…of 10** [*please specify*] |  |  |  |
|  | 1. Order pathology tests (e.g. FBE, Thyroid/Liver function, urine test, serum B12, etc)? |  | **…of 10** [*please specify*] |  |  |  |
|  | 1. Refer for head CT scan? |  | **…of 10** [*please specify*] |  |  |  |
|  | 1. Review current medications being used? |  | **…of 10** [*please specify*] |  |  |  |
|  | 1. Refer to a specialist, either directly, or through a Cognitive, Dementia and Memory Service (CDAMS) or an Aged Care Assessment Service (ACAS)? |  | **…of 10** [*please specify*] |  |  |  |

**^1^** ‘Validated scale’ refers to the use of a standardised scale specifically developed and validated to assess depression in patients with suspected cognitive impairment. For example, the Geriatric Depression Scale (GDS) or the Hamilton Depression Rating Scale. The term ‘validated scale’ does not include the use of non-standardised questions that could be used to make a clinical assessment of depression.

**PART C – Assessing cognitive function using the MMSE**

The questions in this section all relate to **patients 70 years and over with suspected cognitive impairment.**

Please respond to each question below by **circling** a number from 1 to 7 which best reflects your **views about using the Mini Mental State Examination (MMSE) to assess the cognitive function of patients with suspected cognitive impairment.**

**IMPORTANT:** Regardless of whether you use or don’t use the MMSE to assess the cognitive function of patients with suspected cognitive impairment, please STILL answer all of the questions in this section. Your responses will provide useful information about the factors that may influence use of the MMSE in practice.

|  | | | | | | | | | | | | | | | | | | | | | | | | | |  |
| --- | --- | --- | --- | --- | --- | --- | --- | --- | --- | --- | --- | --- | --- | --- | --- | --- | --- | --- | --- | --- | --- | --- | --- | --- | --- | --- |
| **1** | People who are important to me professionally think that I should use the MMSE to assess the cognitive function of patients with suspected cognitive impairment | | | | | ***Strongly disagree*** | | | 1 | | 2 | | 3 | | 4 | | | | 5 | | 6 | | 7 | ***Strongly agree*** | |  |
|  |  | | | | |  | | |  | |  | |  | |  | | | |  | |  | |  |  | |  |
| **2** | For me, using the MMSE to assess the cognitive function of these patients is easy | | | | | ***Strongly disagree*** | | | 1 | | 2 | | 3 | | 4 | | | | 5 | | 6 | | 7 | ***Strongly agree*** | |  |
|  |  | | | | |  | | |  | |  | |  | |  | | | |  | |  | |  |  | |  |
| **3** | I would make it a high priority to use the MMSE to assess the cognitive function of these patients | | | | | ***Strongly disagree*** | | | 1 | | 2 | | 3 | | 4 | | | | 5 | | 6 | | 7 | ***Strongly agree*** | |  |
|  |  | | | | |  | | |  | |  | |  | |  | | | |  | |  | |  |  | |  |
| **4** | It is expected of me that I use the MMSE to assess the cognitive function of these patients | | | | | ***Strongly disagree*** | | | 1 | | 2 | | 3 | | 4 | | | | 5 | | 6 | | 7 | ***Strongly agree*** | |  |
|  |  | | | | |  | | |  | |  | |  | |  | | | |  | |  | |  |  | |  |
| **5** | I am skilled at using the MMSE to assess the cognitive function of these patients | | | | | ***Strongly disagree*** | | | 1 | | 2 | | 3 | | 4 | | | | 5 | | 6 | | 7 | ***Strongly agree*** | |  |
|  |  | | | | |  | | |  | |  | |  | |  | | | |  | |  | |  |  | |  |
| **6** | Lack of time may prevent me from using the MMSE to assess the cognitive function of these patients | | | | | ***Strongly disagree*** | | | 1 | | 2 | | 3 | | 4 | | | | 5 | | 6 | | 7 | ***Strongly agree*** | |  |
|  |  | | | | |  | | |  | |  | |  | |  | | | |  | |  | |  |  | |  |
| **7** | I know how to use the MMSE to assess the cognitive function of these patients | | | | | ***Strongly disagree*** | | | 1 | | 2 | | 3 | | 4 | | | | 5 | | 6 | | 7 | ***Strongly agree*** | |  |
|  |  | | | | |  | | |  | |  | |  | |  | | | |  | |  | |  |  | |  |
| **8** | I have access to the MMSE at my practice to assess the cognitive function of these patients | | | | | ***Strongly disagree*** | | | 1 | | 2 | | 3 | | 4 | | | | 5 | | 6 | | 7 | ***Strongly agree*** | |  |
|  |  | | | | |  | | |  | |  | |  | |  | | | |  | |  | |  |  | |  |
| **9** | I am practised in using the MMSE to assess the cognitive function of these patients | | | | | ***Strongly disagree*** | | | 1 | | 2 | | 3 | | 4 | | | | 5 | | 6 | | 7 | ***Strongly agree*** | |  |
|  | | | | | | | | | | | | | | | | | | | | | | | | | |  |
| **10** | Using the MMSE to assess the cognitive function of these patients is sometimes stressful | | | | | ***Strongly disagree*** | | | 1 | | 2 | | 3 | | 4 | | | | 5 | | 6 | | 7 | ***Strongly agree*** | |  |
|  | | | | | | | | | | | | | | | | | | | | | | | | | |  |
| **11** | My working environment is not conducive to using the MMSE to assess the cognitive function of these patients | | | | | ***Strongly disagree*** | | | 1 | | 2 | | 3 | | 4 | | | | 5 | | 6 | | 7 | ***Strongly agree*** | |  |
|  | | | | | | | | | | | | | | | | | | | | | | | | | |  |
| **12** | People whose opinions I value would approve of me using the MMSE to assess the cognitive function of these patients | | | | | ***Strongly disagree*** | | | 1 | | 2 | | 3 | | 4 | | | | 5 | | 6 | | 7 | ***Strongly agree*** | |  |
|  | | | | | | | | | | | | | | | | | | | | | | | | | |  |
| **13** | I am confident that I can use the MMSE to assess the cognitive function of these patients if I want to | | | | | ***Strongly disagree*** | | | 1 | | 2 | | 3 | | 4 | | | | 5 | | 6 | | 7 | ***Strongly agree*** | |  |
|  | | | | | | | | | | | | | | | | | | | | | | | | | |  |
| **14** | I plan to use the MMSE to assess the cognitive function of these patients | | | | | ***Strongly disagree*** | | | 1 | | 2 | | 3 | | 4 | | | | 5 | | 6 | | 7 | ***Strongly agree*** | |  |
|  | | | | | | | | | | | | | | | | | | | | | | | | | |  |
| **15** | I’m not comfortable using the MMSE to assess the cognitive function of these patients | | | | | ***Strongly disagree*** | | | 1 | | 2 | | 3 | | 4 | | | | 5 | | 6 | | 7 | ***Strongly agree*** | |  |
|  |  | | | | |  | | |  | |  | |  | |  | | | |  | |  | |  |  | |  |
| **16** | I consider myself to be competent in using the MMSE to assess the cognitive function of these patients | | | | | ***Strongly disagree*** | | | 1 | | 2 | | 3 | | 4 | | | | 5 | | 6 | | 7 | ***Strongly agree*** | |  |
|  | | | | | | | | | | | | | | | | | | | | | | | | | |  |
| **17** | Using the MMSE to assess the cognitive function of these patients:  [*circle a response for each item below*] | | | | | | | | | | | | | | | | | | | | | | | | |  |
| **a)** | | ***Is informative*** | 1 | 2 | 3 | | 4 | 5 | | | | 6 | | 7 | | | ***Is uninformative*** | | | | | | | | |  |
| **b)** | | *Is a waste of my time* | 1 | 2 | 3 | | 4 | 5 | | | | 6 | | 7 | | | ***Is a good use of my time*** | | | | | | | | |  |
| **c)** | | *Is necessary* | 1 | 2 | 3 | | 4 | 5 | | | | 6 | | 7 | | | ***Is unnecessary*** | | | | | | | | |  |
| **d)** | | *Leads to good outcomes for the patient* | 1 | 2 | 3 | | 4 | 5 | | | | 6 | | 7 | | | ***Leads to bad outcomes for the patient*** | | | | | | | | |  |
|  | | | | | | | | | | | | | | | | | | | | | | | | | |  |
| **18** | I intend to use the MMSE to assess the cognitive function of these patients | | | | | ***Strongly disagree*** | | | 1 | | 2 | | 3 | | | 4 | | 5 | | 6 | | 7 | | | ***Strongly agree*** | |
|  | | | | | | | | | | | | | | | | | | | | | | | | | | |
| **19** | Using the MMSE to assess the cognitive function of these patients is aversive | | | | | ***Strongly disagree*** | | | 1 | | 2 | | 3 | | | 4 | | 5 | | 6 | | 7 | | | ***Strongly agree*** | |
|  | | | | | | | | | | | | | | | | | | | | | | | | | | |
| **20** | How much do you know about validated scales for assessing cognitive function in these patients? | | | | | ***Nothing*** | | | 1 | 2 | | | 3 | | | 4 | | 5 | | 6 | | 7 | | | ***A great deal­­*** | |
|  | | | | | | | | | | | | | | | | | | | | | | | | | | |
| **21** | How much do you know about the Mini Mental State Examination (MMSE)? | | | | | ***Nothing*** | | | 1 | 2 | | | 3 | | | 4 | | 5 | | 6 | | 7 | | | ***A great deal­­*** | |
|  | | | | | | | | | | | | | | | | | | | | | | | | | | |
| **22** | How familiar are you with clinical guidelines for managing suspected cognitive impairment? | | | | | ***Not at all familiar*** | | | 1 | 2 | | | 3 | | | 4 | | 5 | | 6 | | 7 | | | ***Very familiar*** | |

**PART D – Assessing depression using a validated scale**

The questions in this section all relate to **patients 70 years and over with suspected cognitive impairment.**

Please respond to each question below by **circling** a number from 1 to 7 which best reflects your **views about using a validated scale to assess patients 70 years and over with suspected cognitive impairment for depression.**

The term ‘validated scale’ refers to the use of a standardised scale specifically developed and validated to assess depression in patients with suspected cognitive impairment. For example, the Geriatric Depression Scale (GDS) or the Hamilton Depression Rating Scale. The term ‘validated scale’ does not include the use of non-standardised questions that could be used to make a clinical assessment of depression.

**IMPORTANT:** Regardless of whether you use or don’t use a validated scale to assess patients with suspected cognitive impairment for depression, please STILL answer all of the questions in this section. Your responses will provide useful information about the factors that may influence the use of validated depression scales in practice.

|  | | | | | | | | | | | | | | | | | | | | | | | | | |  |
| --- | --- | --- | --- | --- | --- | --- | --- | --- | --- | --- | --- | --- | --- | --- | --- | --- | --- | --- | --- | --- | --- | --- | --- | --- | --- | --- |
| **1** | For me, using a validated scale to assess these patients for depression is easy | | | | | ***Strongly disagree*** | | | 1 | | 2 | | 3 | | 4 | | | | 5 | | 6 | | 7 | ***Strongly agree*** | |  |
|  | | | | | | | | | | | | | | | | | | | | | | | | | |  |
| **2** | I would make it a high priority to use a validated scale to assess these patients for depression | | | | | ***Strongly disagree*** | | | 1 | | 2 | | 3 | | 4 | | | | 5 | | 6 | | 7 | ***Strongly agree*** | |  |
|  | | | | | | | | | | | | | | | | | | | | | | | | | |  |
| **3** | I am skilled at using a validated scale to assess these patients for depression | | | | | ***Strongly disagree*** | | | 1 | | 2 | | 3 | | 4 | | | | 5 | | 6 | | 7 | ***Strongly agree*** | |  |
|  | | | | | | | | | | | | | | | | | | | | | | | | | |  |
| **4** | Lack of time may prevent me from using a validated scale to assess these patients for depression | | | | | ***Strongly disagree*** | | | 1 | | 2 | | 3 | | 4 | | | | 5 | | 6 | | 7 | ***Strongly agree*** | |  |
|  | | | | | | | | | | | | | | | | | | | | | | | | | |  |
| **5** | Unless I pay attention I may miss the opportunity to use a validated scale to assess these patients for depression | | | | | ***Strongly disagree*** | | | 1 | | 2 | | 3 | | 4 | | | | 5 | | 6 | | 7 | ***Strongly agree*** | |  |
|  | | | | | | | | | | | | | | | | | | | | | | | | | |  |
| **6** | I know how to use a validated scale to assess these patients for depression | | | | | ***Strongly disagree*** | | | 1 | | 2 | | 3 | | 4 | | | | 5 | | 6 | | 7 | ***Strongly agree*** | |  |
|  | | | | | | | | | | | | | | | | | | | | | | | | | |  |
| **7** | I have access to one or more validated scales at my practice to assess these patients for depression | | | | | ***Strongly disagree*** | | | 1 | | 2 | | 3 | | 4 | | | | 5 | | 6 | | 7 | ***Strongly agree*** | |  |
|  |  | | | | |  | | |  | |  | |  | |  | | | |  | |  | |  |  | |  |
| **8** | I am practised in using a validated scale to assess these patients for depression | | | | | ***Strongly disagree*** | | | 1 | | 2 | | 3 | | 4 | | | | 5 | | 6 | | 7 | ***Strongly agree*** | |  |
|  | | | | | | | | | | | | | | | | | | | | | | | | | |  |
| **9** | Using a validated scale to assess these patients for depression is sometimes stressful | | | | | ***Strongly disagree*** | | | 1 | | 2 | | 3 | | 4 | | | | 5 | | 6 | | 7 | ***Strongly agree*** | |  |
|  | | | | | | | | | | | | | | | | | | | | | | | | | |  |
| **10** | Sometimes it’s difficult to decide whether to use a validated scale to assess these patients for depression or not | | | | | ***Strongly disagree*** | | | 1 | | 2 | | 3 | | 4 | | | | 5 | | 6 | | 7 | ***Strongly agree*** | |  |
|  | | | | | | | | | | | | | | | | | | | | | | | | | |  |
| **11** | My working environment is not conducive to using a validated scale to assess these patients for depression | | | | | ***Strongly disagree*** | | | 1 | | 2 | | 3 | | 4 | | | | 5 | | 6 | | 7 | ***Strongly agree*** | |  |
|  | | | | | | | | | | | | | | | | | | | | | | | | | |  |
| **12** | I am confident that I can use a validated scale to assess these patients for depression if I want to | | | | | ***Strongly disagree*** | | | 1 | | 2 | | 3 | | 4 | | | | 5 | | 6 | | 7 | ***Strongly agree*** | |  |
|  | | | | | | | | | | | | | | | | | | | | | | | | | |  |
| **13** | I plan to use a validated scale to assess these patients for depression | | | | | ***Strongly disagree*** | | | 1 | | 2 | | 3 | | 4 | | | | 5 | | 6 | | 7 | ***Strongly agree*** | |  |
|  | | | | | | | | | | | | | | | | | | | | | | | | | |  |
| **14** | I’m not comfortable using a validated scale to assess these patients for depression | | | | | ***Strongly disagree*** | | | 1 | | 2 | | 3 | | 4 | | | | 5 | | 6 | | 7 | ***Strongly agree*** | |  |
|  | | | | | | | | | | | | | | | | | | | | | | | | | |  |
| **15** | I consider myself to be competent in using a validated scale to assess these patients for depression | | | | | ***Strongly disagree*** | | | 1 | | 2 | | 3 | | 4 | | | | 5 | | 6 | | 7 | ***Strongly agree*** | |  |
|  | | | | | | | | | | | | | | | | | | | | | | | | | |  |
| **16** | Using a validated scale to assess patients with suspected cognitive impairment for depression:  [*circle a response for each item below*] | | | | | | | | | | | | | | | | | | | | | | | | |  |
| **a)** | | ***Is informative*** | 1 | 2 | 3 | | 4 | 5 | | | | 6 | | 7 | | | ***Is uninformative*** | | | | | | | | |  |
| **b)** | | *Is a waste of my time* | 1 | 2 | 3 | | 4 | 5 | | | | 6 | | 7 | | | ***Is a good use of my time*** | | | | | | | | |  |
| **c)** | | *Is necessary* | 1 | 2 | 3 | | 4 | 5 | | | | 6 | | 7 | | | ***Is unnecessary*** | | | | | | | | |  |
| **d)** | | *Leads to good outcomes for the patient* | 1 | 2 | 3 | | 4 | 5 | | | | 6 | | 7 | | | ***Leads to bad outcomes for the patient*** | | | | | | | | |  |
|  | | | | | | | | | | | | | | | | | | | | | | | | | |  |
| **17** | I intend to use a validated scale to assess these patients for depression | | | | | ***Strongly disagree*** | | | 1 | | 2 | | 3 | | | 4 | | 5 | | 6 | | 7 | | | ***Strongly agree*** | |
|  | | | | | | | | | | | | | | | | | | | | | | | | | | |
| **18** | Using a validated scale to assess these patients for depression is aversive | | | | | ***Strongly disagree*** | | | 1 | | 2 | | 3 | | | 4 | | 5 | | 6 | | 7 | | | ***Strongly agree*** | |
|  | | | | | | | | | | | | | | | | | | | | | | | | | | |
| **19** | I may forget to use a validated scale to assess these patients for depression | | | | | ***Strongly disagree*** | | | 1 | | 2 | | 3 | | | 4 | | 5 | | 6 | | 7 | | | ***Strongly agree*** | |
|  | | | | | | | | | | | | | | | | | | | | | | | | | | |
| **20** | How much do you know about validated scales for assessing depression in these patients? | | | | | ***Nothing*** | | | 1 | 2 | | | 3 | | | 4 | | 5 | | 6 | | 7 | | | ***A great deal­­*** | |
|  | | | | | | | | | | | | | | | | | | | | | | | | | | |
| **21** | How much do you know about the Geriatric Depression Scale (GDS)? | | | | | ***Nothing*** | | | 1 | 2 | | | 3 | | | 4 | | 5 | | 6 | | 7 | | | ***A great deal­­*** | |

**PART E – Talking to people about the diagnosis**

The questions in this section all relate to **situations where you are confident of the diagnosis of dementia.**

Disclosure of diagnosis is a complex process. The questions in this section cover some of the actions within that process including:

- finding out what the patient already knows or suspects about their diagnosis
- using dementia-specific words when talking to the patient
- exploring what the diagnosis means to the patient
- involving family members or carers in the disclosure process

Please respond to each question below by **circling** a number from 1 to 7 which best reflects your **views about talking to people and their carers about the diagnosis.**

| **1** | **I intend to....** [*circle a response for each item below*] | | | | | | | | | | |
| --- | --- | --- | --- | --- | --- | --- | --- | --- | --- | --- | --- |
| 1. find out what the patient already knows or suspects about their diagnosis | | ***Strongly disagree*** | 1 | 2 | 3 | 4 | 5 | 6 | 7 | ***Strongly agree*** |  |
| 1. use the actual words ‘dementia’, or the specific type of dementia (e.g. ‘Alzheimer’s disease’ etc) when talking to the patient | | ***Strongly disagree*** | 1 | 2 | 3 | 4 | 5 | 6 | 7 | ***Strongly agree*** |  |
| 1. explore what the diagnosis means to the patient | | ***Strongly disagree*** | 1 | 2 | 3 | 4 | 5 | 6 | 7 | ***Strongly agree*** |  |
| 1. involve the appropriate family member/carer in the disclosure of diagnosis by ensuring they know what the patient has been told about their diagnosis | | ***Strongly disagree*** | 1 | 2 | 3 | 4 | 5 | 6 | 7 | ***Strongly agree*** |  |
| **2** | **In my practice I expect to....** [*circle a response for each item below*] | | | | | | | | | | |
| 1. find out what the patient already knows or suspects about their diagnosis | | ***Strongly disagree*** | 1 | 2 | 3 | 4 | 5 | 6 | 7 | ***Strongly agree*** |  |
| 1. use the actual words ‘dementia’, or the specific type of dementia (e.g. ‘Alzheimer’s disease’, ‘vascular dementia’, etc) when talking to the patient | | ***Strongly disagree*** | 1 | 2 | 3 | 4 | 5 | 6 | 7 | ***Strongly agree*** |  |
| 1. explore what the diagnosis means to the patient | | ***Strongly disagree*** | 1 | 2 | 3 | 4 | 5 | 6 | 7 | ***Strongly agree*** |  |
| 1. involve the appropriate family member/carer in the disclosure of diagnosis by ensuring they know what the patient has been told about their diagnosis | | ***Strongly disagree*** | 1 | 2 | 3 | 4 | 5 | 6 | 7 | ***Strongly agree*** |  |

**Thank you very much for your participation!**

Please return the survey in the reply-paid envelope provided.

**THIS PAGE HAS BEEN LEFT INTENTIONALLY BLANK**
