# Supplementary material for: Evidence-based care of older people with suspected cognitive impairment in general practice: protocol for the IRIS cluster randomised trial
Source: Implement Sci. 2013 Aug 19;8:91. doi: 10.1186/1748-5908-8-91 (PMC3765181; doi:10.1186/1748-5908-8-91)
Supplement: Additional file 2 — IRIS sample size calculations. This file provides details of the sample size calculations used in IRIS. [file 1748-5908-8-91-S2.docx]

## Additional file 2 – IRIS Sample size calculations

The primary outcomes of the IRIS trial include *cognitive assessment using MMSE* and *depression assessment using a validated scale* in patients with suspected cognitive impairment (cohort 2). In the calculation of sample size for these outcomes, adjustment needs to be made for the clustered nature of the design. The variance inflation factor, or design effect, used to achieve this is a function of the average cluster size, variation in cluster size, and the intra-cluster correlation (ICC) [[1](#_ENREF_1)]. Empirical research has suggested ICCs of the order 0.10 for measures of professional behaviour (process measures) [[2](#_ENREF_2)]. The variation in cluster size is incorporated into the sample size calculation through an estimate of the coefficient of variation (CV), defined as the ratio of the standard deviation of the cluster sizes to the mean cluster size [[1](#_ENREF_1)]. Estimates of CV that have been observed in general practice in the United Kingdom range from 0.42 – 0.75 [[1](#_ENREF_1)]. We have assumed a CV at the higher end of the range, 0.7, to provide a more conservative estimate of the required sample size.

We had little data on the rates of the behaviours, and so assumed a control group rate of 50% (for both outcomes) in the sample size calculation; this assumption provides the most conservative scenario for the calculated sample size. A Cochrane systematic review investigating the effects of continuing education meetings and workshops of professional practice [[3](#_ENREF_3)], found the median adjusted risk difference in compliance with desired practice was 6% (interquartile range 1.8 to 15.9) when any intervention in which educational meetings were a component was compared to no intervention. This effect was modified by whether the educational sessions were didactic, interactive, or both, with some evidence suggesting that mixed interactive and didactic sessions were more effective (median adjusted risk difference of 13.6%) compared with didactic meetings. We therefore determined that a difference of at least 15% could be achieved, and would be of clinical importance.

Assuming 20 patients per practice (and the parameters above), 45 practices per group will be sufficient to detect the 15% increase in recommended behaviour with 90% power (two-sided significance level of 5%). This will provide outcome data on 900 patients per arm (equivalent to an effective sample size of 232). Allowing for 25% attrition in practices (based on attrition rates in a previous implementation trial we have undertaken in general practice [[4](#_ENREF_4)]), we will aim to recruit 60 practices per group. The width of the 95% confidence interval for an observed difference in rates of 15% (i.e. 50% vs 65%) will be approximately ±9%. On the log odds scale, this is equivalent to ±0.37 (Table 1). The width of the 95% confidence interval for a range of potentially observed risk differences (and corresponding odds ratios), across different control group rates, and numbers of patients per practice, do not modify the width of the confidence intervals importantly (Table 1).

**Table 1: Sensitivity of the width of 95% confidence intervals (of observed intervention effects) to different sample size parameters**

| N per practice^†^ | Control group rate | | Width of 95% confidence intervals for observed differences in rates and corresponding log odds^*^ | | | | | | | |
| --- | --- | --- | --- | --- | --- | --- | --- | --- | --- | --- |
|  |  | *RD = 10%* | | *ln(OR) = 0.44* |  | *RD = 15%* | *ln(OR) =0.65* |  | *RD = 20%* | *ln(OR) = 0.85* |
| 10 | 30% | ±10% | | ±0.43 |  | ±10% | ±0.42 |  | ±10% | ±0.42 |
| 20 |  | ±9% | | ±0.38 |  | ±9% | ±0.38 |  | ±9% | ±0.38 |
| 30 |  | ±8% | | ±0.37 |  | ±8% | ±0.37 |  | ±8% | ±0.37 |
| 40 |  | ±8% | | ±0.36 |  | ±8% | ±0.36 |  | ±8% | ±0.36 |
|  |  |  | |  |  |  |  |  |  |  |
|  |  | *RD = 10%* | | *ln(OR) =0.41* |  | *RD = 15%* | *ln(OR) = 0.61* |  | *RD = 20%* | *ln(OR) =0.81* |
| 10 | 40% | ±10% | | ±0.41 |  | ±10% | ±0.41 |  | ±10% | ±0.41 |
| 20 |  | ±9% | | ±0.37 |  | ±9% | ±0.37 |  | ±9% | ±0.37 |
| 30 |  | ±9% | | ±0.35 |  | ±9% | ±0.35 |  | ±9% | ±0.36 |
| 40 |  | ±8% | | ±0.35 |  | ±8% | ±0.35 |  | ±8% | ±0.35 |
|  |  |  | |  |  |  |  |  |  |  |
|  |  | *RD = 10%* | | *ln(OR) = 0.41* |  | *RD = 15%* | *ln(OR) = 0.62* |  | *RD = 20%* | *ln(OR) = 0.85* |
| 10 | 50% | ±10% | | ±0.41 |  | ±10% | ±0.41 |  | ±10% | ±0.42 |
| 20 |  | ±9% | | ±0.37 |  | **±9%‡** | **±0.37‡** |  | ±9% | ±0.38 |
| 30 |  | ±9% | | ±0.35 |  | ±9% | ±0.36 |  | ±8% | ±0.37 |
| 40 |  | ±8% | | ±0.35 |  | ±8% | ±0.35 |  | ±8% | ±0.36 |
|  |  |  | |  |  |  |  |  |  |  |

RD = risk difference; ln(OR) = log odds ratio

^*^ Width of confidence intervals calculated assuming the number of practices is fixed (i.e. 45 practices per intervention group), and varying other sample size parameters (number of patients per practice, control group rate of recommended behaviour, and observed effect size (either difference in rates or difference in log odds).

^†^ Number of patients per practice.

‡ Sample size based on this combination of parameters.

## References

1. Eldridge SM, Ashby D, Kerry S: **Sample size for cluster randomized trials: effect of coefficient of variation of cluster size and analysis method**. *International journal of epidemiology* 2006, **35**(5):1292-1300.

2. Campbell M, Grimshaw J, Steen N: **Sample size calculations for cluster randomised trials. Changing Professional Practice in Europe Group (EU BIOMED II Concerted Action)**. *J Health Serv Res Policy* 2000, **5**(1):12-16.

3. Forsetlund L, Bjorndal A, Rashidian A, Jamtvedt G, O'Brien MA, Wolf F, Davis D, Odgaard-Jensen J, Oxman AD: **Continuing education meetings and workshops: effects on professional practice and health care outcomes**. *Cochrane Database Syst Rev* 2009(2):CD003030.

4. McKenzie JE, French SD, O'Connor DA, Grimshaw JM, Mortimer D, Michie S, Francis J, Spike N, Schattner P, Kent PM *et al*: **IMPLEmenting a clinical practice guideline for acute low back pain evidence-based manageMENT in general practice (IMPLEMENT): cluster randomised controlled trial study protocol**. *Implement Sci* 2008, **3**:11.
