# Supplementary material for: Evidence-based care of older people with suspected cognitive impairment in general practice: protocol for the IRIS cluster randomised trial
Source: Implement Sci. 2013 Aug 19;8:91. doi: 10.1186/1748-5908-8-91 (PMC3765181; doi:10.1186/1748-5908-8-91)
Supplement: Additional file 3 — IRIS additional methods for the economic evaluation. This file provides additional details of the methods used for the economic evaluation alongside the IRIS trial. [file 1748-5908-8-91-S3.docx]

### Additional file 3 – IRIS Additional methods for the economic evaluation

The intervention and comparator, perspective and time horizon for the cost-effectiveness analysis alongside the IRIS trial are described in the main text. Additional methods for the IRIS CEA including the identification, measurement and valuation of costs and consequences, adjustment for differential timing and methods for summarising decision uncertainty are described below.

**Identification, measurement, and valuation of health outcomes**

Banjeree and Wittenberg (2009) identified a number of health benefits associated with early diagnosis and timely intervention for dementia including improvements in quality of life associated with diversion from residential care and treatment of common comorbidities including depression. Banjeree and Wittenberg (2009) also recognise the potential for “negative reactions to diagnosis” but conclude that “the balance is very much in favour of diagnosis and the earlier in the illness the better”. The trial-based economic evaluation described here is not designed to quantify treatment effects and cost-effectiveness in terms of the final health benefits and disbenefits. Rather, we rely on evidence from previous studies (see Banerjee & Wittenberg, 2009) that the health benefits of early detection and diagnosis of dementia and common comorbidities outweigh any disbenefits and treat adherence to recommended behaviours for early detection and diagnosis as surrogates for final health outcomes.

In line with the main analysis, the primary outcomes for the economic evaluation alongside the IRIS trial will be cognitive assessment using MMSE and depression assessment using validated scale (based on review of application of the CAT to GP medical records). Intervention effects with respect to these primary outcomes will be estimated using methods specified for the main analysis and expressed as between-group differences in the number of patients assessed using MMSE and the number of patients receiving depression assessment by applying risk differences to the median number of patients per GP meeting the criteria for membership of cohort 2 within the trial population.

**Identification, measurement, and valuation of resource use**

Incremental costs will reflect resource use associated with delivery of the implementation intervention, and any changes in clinical practice with respect to detection and diagnosis of dementia observed within the trial period. Cost items associated with delivery of the IRIS implementation intervention – as distinct from development of the implementation intervention (Mortimer et al, 2008) – include administrative costs associated with coordinating the intervention activities; labour/consumables required for delivery of the face-to-face workshop including attendance and preparation time for opinion leaders, facilitators and GP participants; opportunity cost of venue for workshops; production and distribution of DVDs. Cost items associated with delivery of the control condition include administrative costs associated with coordinating the control activities; and labour/consumables required for production and distribution of the CPG to control GPs.

Costs arising from changes in clinical practice include the costs of clinical investigations including conduct of MMSE, screening for comorbid depression using a validated tool, pathology testing and CT scanning. Other costs arising from change in clinical practice include use of prescription pharmaceuticals and use of services provided by CDAMS, ACAS or specialists for dementia and/or co-morbid conditions such as depression. With the exception of use of prescription pharmaceuticals, resource use arising from changes in clinical practice will be reflected in the secondary outcomes described in Table 2 of the main text.

Costs arising from changes in health status include the costs(savings) of diversion from residential care, decreased use of primary care and community social care, and prevention or early discharge from acute hospital admissions (Banerjee & Wittenberg, 2009). Costs arising from changes in health status cannot be explicitly included in the cost analysis (as a consequence of the time horizon and the measurement of outcomes via the application of the CAT to GP medical records) but may be reflected in the ‘surrogate outcome’ of adherence to the key recommendations of the CPG.

Resource use associated with the delivery of the implementation strategy will be estimated from administrative and financial records detailing costs associated with the production and distribution of materials including workshop DVDs; total person hours spent in organising and facilitating face-to-face workshops; duration of and attendance at workshops; venue location and total hours venue use for workshops. Resource use associated with a change in clinical practice will be based on CAT-based measurement of the primary and secondary outcomes described in Table 2 of the main text.

Unit costs for routinely provided health services including GP consultations, specialist services, and CT scans will be as per the Manual of Resource Items for use in submissions to the Commonwealth of Australia's Pharmaceutical Benefits Advisory Committee (PES, 2009). Goods and services within the health sector but not included in the manual will be valued based on the actual average cost of delivery obtained from published sources where available or on market prices for identical or similar products where market prices reflect the opportunity cost of resource use. For example, unit costs for referral to CDAMS will be based on estimates of average cost per client inflated to AUD for the year of study completion (Foreman et al, 2003). In the case of the direct costs of the intervention associated with, for example, use of meeting rooms for delivery of the intervention and the cost of materials, unit costs will be obtained either from administrative or financial records documenting the actual price paid or from advertised prices of similar products.

Total cost for each individual GP will be obtained as the summation over the GP-level data regarding total delivery cost and patient-level data regarding changes in the clinical practice of each GP. Given the characteristic distribution of health costs (truncated at zero and right skewed), the importance of obtaining readily interpretable marginal effects, and our interest in population-average effects, we will model intervention effects on total costs using one-part GEEs with a log link rather than transformed ordinary least squares or two-part models (Buntin & Zaslavsky, 2004). Specification of the log link for the GEE model permits natural interpretation of marginal effects on cost without retransformation (Buntin & Zaslavsky, 2004). Correlation structure, standard errors and controls for confounding variables will be as specified for GP behaviours in the main analysis.

**Adjustment for differential timing**

All costs will be inflated to current AUD for the year of study completion. All costs and benefits will be converted to present values for the year of study completion using an annual discount rate of 5% in the base-case, and annual rates of 3% and 7% in sensitivity analysis.

**Uncertainty**

Cost-effectiveness acceptability curves (CEACs) will be derived from the joint density of incremental costs (ΔC) and incremental effects (ΔE) for the intervention as compared to the comparator of passive dissemination. The joint density will be obtained via non-parametric bootstrapping from the distribution of observed cost/effect pairs for patient participants. Separate CEACs will be derived for upper/lower bound estimates of a pre-specified set of uncertain parameters not estimated with sampling error: unit cost for ED staff time (AUD 0, average hourly wage rate) and the discount rate (3%, 7%).

**References**

Banerjee S, Wittenberg R. (2009) **Clinical and cost effectiveness of services for early diagnosis and intervention in dementia.** *Int J Geriatr Psychiatry*, 24(7): 748-54.

Buntin MB, Zaslavsky AM: **Too much ado about two-part models and transformation? Comparing methods of modeling Medicare expenditures**. *J Health Econ* 2004, **23**(3):525-542.

Foreman P, Davis S, Gardner I, Rosewarne R (2003). **Review of the Cognitive, Dementia and Memory Service Clinics, Final Report.** Victorian Department Of Human Services, Melbourne. http://www.health.vic.gov.au/subacute/cdamsrpt2003.pdf

Gilley DW, Bienias JL, Wilson RS, et al. 2004. **Influence of behavioral symptoms on rates of institutionalization for persons with Alzheimer’s disease**. Psychologic Med 34: 1129–1135.

Mortimer D, French SD, McKenzie JE, O'Connor DA, Green SE: **Protocol for economic evaluation alongside the IMPLEMENT cluster randomised controlled trial.** *Implement Sci* 2008, **3:**12.

(PES) PES: **Manual of resource items and their associated costs for use in major submissions to the Pharmaceutical Benefits Advisory Committee involving economic analyses (Version 4.0)**. In*.* Edited by Australia Co; December 2009.
